# Effects of Metabolites Derived from Guava (*Psidium guajava* L.) Leaf Extract Fermented by *Limosilactobacillus fermentum* on Hepatic Energy Metabolism via SIRT1-PGC1α Signaling in Diabetic Mice

**DOI:** 10.3390/nu17010007

**Published:** 2024-12-24

**Authors:** Sohyun Jeon, Heaji Lee, Sun-Yeou Kim, Choong-Hwan Lee, Yunsook Lim

**Affiliations:** 1 Department of Food and Nutrition, Kyung Hee University, 26 Kyunghee-Daero, Dongdaemun-Gu, Seoul 02447, Republic of Korea; rabbitjeon@khu.ac.kr (S.J.); ji3743@khu.ac.kr (H.L.); 2 College of Pharmacy, Gachon University, Incheon 21936, Republic of Korea; sunnykim@gachon.ac.kr; 3 Department of Bioscience and Biotechnology, Konkuk University, Seoul 05029, Republic of Korea; chlee123@konkuk.ac.kr

**Keywords:** type 2 diabetes mellitus, metabolic dysfunction-associated fatty liver disease, energy metabolism, probiotic fermentation, metabolites

## Abstract

Background/Objectives: Type 2 diabetes mellitus (T2DM) is considered a serious risk to public health since its prevalence is rapidly increasing worldwide despite numerous therapeutics. Insulin resistance in T2DM contributes to chronic inflammation and other metabolic abnormalities that generate fat accumulation in the liver, eventually leading to the progression of metabolic dysfunction-associated fatty liver disease (MAFLD). Recently, the possibility that microbial-derived metabolites may alleviate MAFLD through enterohepatic circulation has emerged, but the underlying mechanism remains unclear. In this research, we utilized metabolites obtained from the fermentation of guava leaf extract, which is well-known for its antidiabetic activity, to investigate their effects and mechanisms on MAFLD. Methods: Diabetes was induced by a high-fat diet and streptozotocin injection (80 mg/kg body weight) twice in mice. Subsequently, mice whose fasting blood glucose levels were measured higher than 300 mg/dL were administered with metabolites of *Limosilactobacillus fermentum* (LF) (50 mg/kg/day) or guava leaf extract fermented by *L. fermentum* (GFL) (50 mg/kg/day) by gavage for 15 weeks. Results: GFL supplementation mitigated hyperglycemia and hepatic insulin resistance. Moreover, GFL regulated abnormal hepatic histological changes and lipid profiles in diabetic mice. Furthermore, GFL enhanced energy metabolism by activating the sirtuin1 (SIRT1)/proliferator-activated receptor γ coactivator 1α (PGC1α)/peroxisome proliferator-activated receptor (PPAR)-α pathway in diabetic mice. Meanwhile, GFL supplementation suppressed hepatic inflammation in diabetic mice. Conclusions: Taken together, the current study elucidated that GFL could be a potential therapeutic to ameliorate hyperglycemia and hepatic steatosis by improving SIRT1/PGC-1α/ PPAR-α-related energy metabolism in T2DM.

## 1. Introduction

Type 2 diabetes mellitus (T2DM) is considered a major health concern worldwide since its prevalence is predicted to increase by 12.2% (783 million people) by 2045 [1]. Patients with T2DM have a significantly higher risk of complications such as retinopathy, end-stage renal disease, liver disease, and cardiovascular diseases than people without T2DM [2]. In particular, metabolic dysfunction-associated fatty liver disease (MAFLD) is estimated to affect 55.5% of all T2DM patients and is closely linked to other complications, including cardiovascular disease and hepatocellular carcinoma [3,4].

Patients with T2DM are prone to hepatic steatosis, as hepatic insulin resistance is a primary factor in the pathogenesis of T2DM and MAFLD [5]. Hepatic de novo lipogenesis is a critical pathway in the development of fatty liver [6]. Hyperinsulinemia caused by insulin resistance activates sterol regulatory element-binding protein-1c (SREBP-1c), which increases the expression of genes integral to lipogenesis [5,7].

Dysregulated lipid homeostasis is closely linked to reduced energy metabolism in T2DM [8]. Sirtuin 1 (SIRT1) is a key regulator that controls various metabolic functions by deacetylating various molecules [8]. SIRT1 negatively regulates lipogenic activity by binding to peroxisome proliferator-activated receptor (PPAR)-γ and activates PPAR-α through PGC-1α deacetylation. Activation of PGC-1α leads to enhanced mitochondrial biogenesis and fatty acid oxidation [9]. Hyperglycemia impairs the expression of SIRT1 by depletion of cellular NAD+ [10]. The imbalance between fatty acid oxidation and lipogenesis due to decreased SIRT1 activity in T2DM can lead to hepatic steatosis [8]. Therefore, activation of energy metabolism via the SIRT1/PGC-1α/PPAR-α pathway can be a therapeutic target for MAFLD.

Probiotics, especially lactic acid bacteria, have been used to treat MAFLD for over a decade [11]. Among them, *Limosilactobacillus fermentum* is known to ameliorate fatty liver by regulating lipid metabolism, inflammation, insulin resistance, and oxidative stress in obese mice [12,13,14]. In particular, metabolites derived from *L. fermentum* could be the driving force behind the health benefits it provides. However, limited studies related to MAFLD have focused on the effects and mechanisms of microbial-derived metabolites [15,16,17]. Branched-chain hydroxy acids (BCHAs), one of the metabolites from *L. fermentum*-derived bacteria, are known to have both antiglycation and antioxidant activities in vitro [18]. Recent studies showed that plasma BCHA levels were reduced in obesity, and supplementation of BCHAs prevented insulin resistance and alleviated hepatic steatosis in obese mice [19,20].

Recently, natural products bioconverted by microbes have been utilized for therapeutic purposes, as they can produce various bioactive compounds through the biotransformation of phenolic compounds and microbial metabolites [21,22]. In this study, *Psidium guajava Linn*. (guava tree) [23] was used, as its leaf is well-known to possess potential pharmacologic activities, including antimicrobial [24], antioxidant [25], anti-inflammatory [26], and antidiabetic properties [27]. In our previous study, guava leaf extracts fermented by *L. fermentum* KCTC15072BP showed significantly higher antioxidant and antiglycation activities than those without guava leaf extract in vitro [18]. Guava leaf extract fermented by *L. fermentum* (GFL) is distinguished from metabolites of *L. fermentum* (LF) in that it contains guava leaf extract-derived metabolites, including flavonoid and phenolic acid, as well as bacterial metabolites [28,29]. These prior studies indicate that LF and GFL have the potential to be developed as promising interventions for the treatment of metabolic diseases, including T2DM and MAFLD.

This study tried to investigate the mechanism by which the administration of guava leaf extract fermented by *L. fermentum* ameliorates MAFLD in diabetic mice.

## 2. Materials and Methods

### 2.1. Preparation of Fermented Guava Leaf Extract

Dried guava leaf was purchased from Jeju Island, Korea. In brief, dried guava leaf was extracted with 50% ethanol at room temperature and finally freeze-dried to obtain a powder form of guava leaf extract. Guava leaf extract was added to MRS broth to reach 0.5% (*w/v*) concentration for bioconversion, and MRS broth alone was prepared as a control. The optical density of *Limosilactobacillus fermentum* KCTC (Korean Collection for Type Cultures) 150720BP was adjusted to the value 1.0 (approximately 2.02 × 10^9^ CFU/mL) via spectrophotometry at 600 nm. The bacterial culture was inoculated into a media supplemented with and without 0.5% (*w*/*v*) guava leaf extract to achieve 5% (*v/v*) of the final volume in the media. After 24 h of inoculation, the culture media were harvested and then centrifuged at 11,001× *g* for 10 min at 4 °C. Subsequently, extracellular metabolites were extracted from the supernatant and then dried completely using a speed vacuum (200 rpm at 25 °C for 2 h on a rotary shaker). Metabolites from media with and without guava leaf extract supplement were used in an animal study as GFL and LF, respectively [18].

### 2.2. Experimental Design

The induction of diabetes mellites is described in our previous research [30]. Four-week-old C57BL/6 male mice were purchased from Raon Bio (Yongin, Gyeonggi-do, Republic of Korea) and housed two or three per cage at 22 ± 1 °C temperature, 50 ± 5% humidity, and a 12-h light/dark cycle. Following a one-week acclimation period, six mice were randomly allocated to the normal control (NC) group, receiving a 10% kcal control diet (D12450J; matching sucrose to D12492; Research Diets, New Brunswick, NJ, USA) throughout the entire experiment. The remaining mice received a 60% kcal high-fat diet (D12492; Research Diets, New Brunswick, NJ, USA) and served as the diabetic (DM) group. The food and distilled water were provided ad libitum. After being fed each diet for 4 weeks, the DM group was intraperitoneally injected with 80 mg/kg body weight streptozotocin (Sigma Aldrich, St. Louis, MO, USA) in citrate buffer (pH 4.5) twice, 1 week apart, while the NC group was injected with citrate buffer only. Until 3 weeks from the last injection, mice whose fasting blood glucose (FBG) levels were measured higher than 300 mg/dL at least twice were considered a diabetic state. After induction of diabetes, all mice were divided into four groups and supplemented as follows: (A) the NC group (*n* = 6), non-diabetic mice, was supplemented with distilled water; (B) the diabetic control (DMC) group (*n* = 6), diabetic mice, was supplemented with distilled water; (C) the LF group (*n* = 6), diabetic mice, was supplemented with metabolites of *L. fermentum* (LF) (50 mg/kg body weight/day); and (D) the GFL group (*n* = 5) was supplemented with guava leaf extract fermented by *L. fermentum* (GFL) (50 mg/kg body weight/day). The conversion of LF or GFL dose from mice to humans was calculated to be 4.05 mg/kg [31]. The sample size used in this study was a general sample size for animal experiments, and it can be assumed that it follows a normal distribution [32]. We have assigned mice to each group purely randomly and confirmed that there were no statistical differences between groups. These supplements were dissolved in distilled water and administered by oral gavage every day for 15 weeks. The body weight and food intake were monitored once a week throughout the study period. Also, 8 h fasting blood glucose (FBG) levels from the tail vein were measured every week using a HealthPro blood glucometer (Osang Healthcare, Yongin, Gyeonggi-do, Republic of Korea). The mice were anesthetized using ketamine injection. Blood samples were collected from a cardiac punch and centrifuged at 1945× *g* for 15 min to obtain plasma. Blinding was used for each step of the experimental process. There were no exclusions in this experiment. This experiment was approved by the Institutional Animal Care and Use Committee of Kyung Hee University [KHSASP-23-153, approval date: 15 May 2023] and was performed following the guidelines.

### 2.3. Hemoglobin A1c

Before the mice were sacrificed, hemoglobin A1c (HbA1c) level was measured by Clover A1c Analyzer (Infopia Co., Ltd., Anyang, Gyeonggi-do, Republic of Korea) using 4 μL blood from the tail vein.

### 2.4. Hepatic Histological Analysis

Liver tissues, fixed in a 10% formalin solution, were paraffin-embedded for histological observations. Tissues were sectioned to a thickness of 4 μm each and examined using an optical microscope (Olympus Optical, Tokyo, Japan). The size, number, and area of hepatic lipid droplets were analyzed using Image J software (version 1.54, NIH, Bethesda, MD, USA).

### 2.5. Hepatic Lipid Profile Analysis

Triglyceride (TG) and total cholesterol (TC) levels in the liver were determined using commercially available assay kits from Asan Pharmaceutical. The extraction of hepatic lipids was carried out using the Folch method, involving a 2:1 chloroform/methanol mixture [33].

### 2.6. Protein Extraction and Western Blot Analysis

The protein extraction and quantification of the liver were performed according to a method previously described [34]. For each group, 30 μg of protein was prepared for the western blot. The proteins were separated via 6–12% SDS-PAGE and then transferred to a polyvinylidene fluoride (PVDF) membrane (Millipore, Billerica, MA, USA). Membranes were incubated overnight with primary antibodies, followed by treatment with corresponding secondary antibodies (Biorad, Hercules, CA, USA). All quantifications were normalized to the levels of α-tubulin (cytosol) or PCNA (nucleus) [34].

The following primary antibodies were used: p-IRS-1 (Ser302), IRS-1 (Cell signaling Technology, Danvers, MA, USA, 1:200), NF-kB, interleukin-1β (IL-1β), p-ACC, ACC (Cell signaling Technology, MA, USA, 1:2000), PGC-1α, SREBP-1c, PPAR-α, PPAR-γ, SIRT1, SIRT4, p-Akt (Ser473), Akt, tumor necrosis factor-α (TNF-α), IL-6 (Santa Cruz Biotechnology, CA, USA, 1:200), PCNA (Enzolife markers science, Long island, NY, USA. 1:2000), and α-tubulin (Sigma Aldrich, St. Louis, MO, USA, 1:5000)

### 2.7. Statistical Analysis

All data were presented as means ± standard deviation (SD) and were statistically analyzed using SPSS software (version 23.0 for Windows, SPSS Inc., Chicago, IL, USA). Differences among the means of all parameters were evaluated through a one-way analysis of variance (ANOVA), followed by Duncan’s multiple range test. A *p*-value < 0.05 was considered statistically significant.

## 3. Results

### 3.1. Effects of GFL Supplementation on Body Weight, Food Intake, and Liver Index in Diabetic Mice

Food intake in the GFL group was significantly lower than that in the DMC group. Liver weight normalized against body weight revealed a significant difference between the NC group and the DM groups. In particular, liver weight was significantly lower in the GFL group than in the DMC group (Table 1).

### 3.2. Effects of GFL Supplementation Fasting Blood Glucose and Hemoglobin A1c Levels in Diabetic Mice

**Figure 1:**
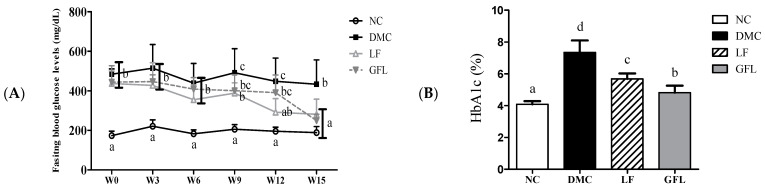
GFL supplementation decreased (**A**) fasting blood glucose and (**B**) hemoglobin A1c (HbA1c) levels in diabetic mice. All values above are represented as mean ± SD (n = 5–6). Means with different letters were significantly different. A *p*-value < 0.05 was considered statistically significant.

FBG levels in the DMC group exhibited higher than those in the NC group before they were administered any supplementation. However, after receiving supplements for 15 weeks, FBG levels in the LF and GFL groups were significantly lower than in the DMC group (Figure 1A). Similarly, HbA1c in both LF and GFL groups showed a significant decrease compared to that of the DMC group (Figure 1B).

### 3.3. Effects of GFL Supplementation on Hepatic Histology in Diabetic Mice

**Figure 2:**
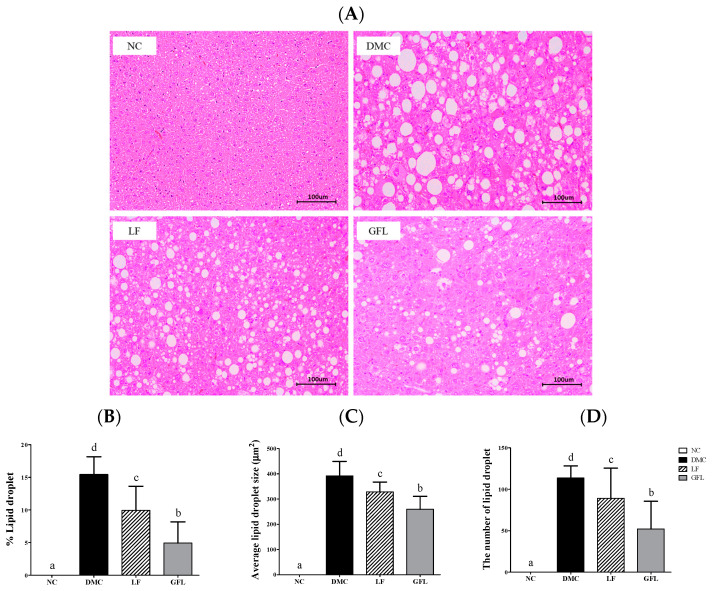
GFL supplementation alleviated (**A**) hepatic morphology (200× magnification) in diabetic mice. (**B**) % lipid droplet, (**C**) average lipid droplet size, and (**D**) the number of lipid droplets. All values above are represented as mean ± SD (n = 3). Means with different letters were significantly different. A *p*-value < 0.05 was considered statistically significant.

The percentage area, average size, and number of lipid droplets in the DMC group were increased compared to those of the NC group (Figure 2A–D). After LF and GFL supplementation, these parameters were reduced compared to the DMC group. In particular, all parameters were lower in the GFL group compared to the LF group.

### 3.4. Effects of GFL Supplementation on Hepatic Lipid Profiles in Diabetic Mice

**Figure 3:**
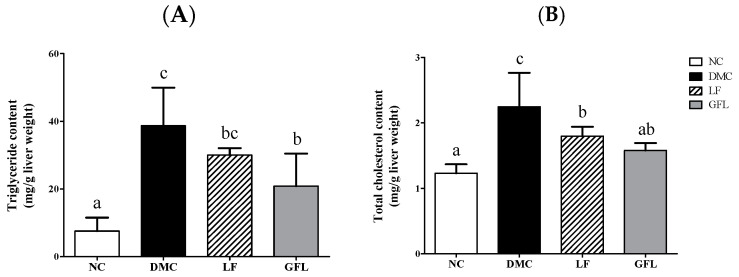
GFL supplementation mitigated hepatic lipid profiles in diabetic mice. (**A**) triglyceride content and (**B**) total cholesterol content All values above are represented as mean ± SD (n = 5–6). Means with different letters were significantly different. A *p*-value < 0.05 was considered statistically significant.

The hepatic TG level was significantly decreased in the GFL group compared to that of the DMC group (Figure 3A). However, hepatic TG level was not changed by LF supplementation in the diabetic mice. On the other hand, the hepatic TC level was significantly lower in the LF and GFL groups than in the DMC group (Figure 3B).

### 3.5. Effects of GFL Supplementation on Hepatic Insulin Signaling in Diabetic Mice

**Figure 4:**
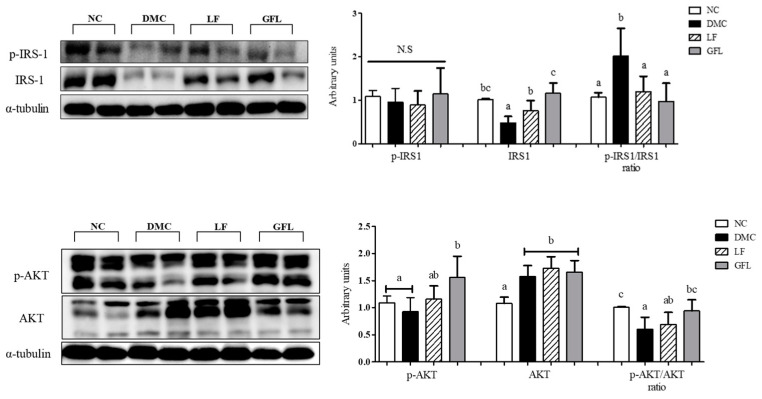
GFL supplementation improved hepatic insulin signaling in diabetic mice. All values above are represented as mean ± SD (n = 5–6). Means with different letters were significantly different. A *p*-value < 0.05 was considered statistically significant.

The level of p-IRS-1/IRS ratio indicating insulin resistance was significantly higher in the DMC group than in other groups. The level of p-IRS-1/IRS ratio was markedly reduced in the LF and GFL groups compared to the DMC group (Figure 4). The level of the p-AKT/AKT ratio was higher in the GFL group than in the DMC group.

### 3.6. Effects of GFL Supplementation on Hepatic Lipid Metabolism in Diabetic Mice

**Figure 5:**
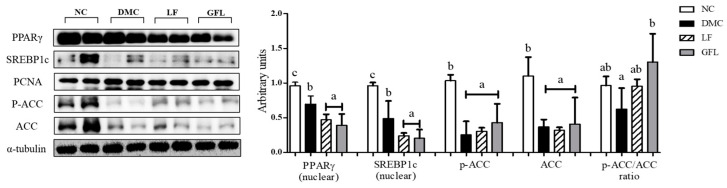
GFL supplementation reduced hepatic lipogenesis in diabetic mice. All values above are represented as mean ± SD (n = 5–6). Means with different letters were significantly different. A *p*-value < 0.05 was considered statistically significant.

The protein levels of PPAR-γ and SREBP-1c were decreased in the LF and GFL groups compared to those of the DMC group. The level of p-ACC/ACC ratio in the GFL group was significantly increased compared to that of the DMC group (Figure 5).

### 3.7. Effects of GFL Supplementation on Hepatic Energy Metabolism in Diabetic Mice

**Figure 6:**
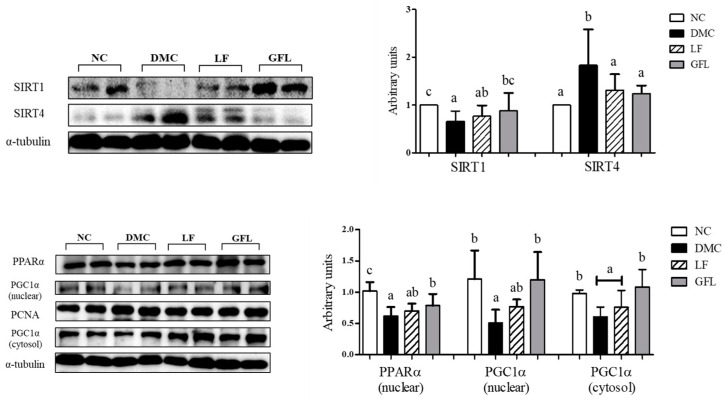
GFL supplementation enhanced hepatic energy metabolism in diabetic mice. All values above are represented as mean ± SD (n = 5–6). Means with different letters were significantly different. A *p*-value < 0.05 was considered statistically significant.

The protein levels of SIRT1, PPAR-α, and PGC-1α showed consistent statistical results among the treatment groups, in which the levels in the DMC group were lowered compared to those in the NC group, while the levels in the GFL group were higher than those in the DMC group. In particular, PGC-1α showed consistent results regardless of its location (nuclear or cytoplasmic) (Figure 6).

The protein level of SIRT4, which interacts oppositely with SIRT1, showed a significant increase in the DMC group compared to that of the NC group. The protein level of SIRT4 was significantly restrained in the LF and GFL groups compared to the DMC group (Figure 6).

### 3.8. Effects of GFL Supplementation on Hepatic Inflammation in Diabetic Mice

**Figure 7:**
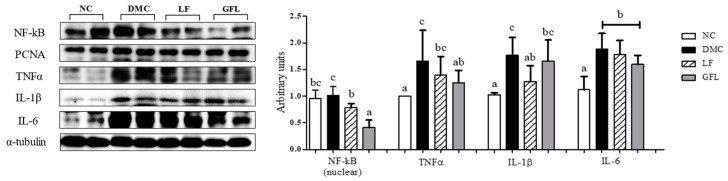
GFL supplementation attenuated hepatic inflammation in diabetic mice. All values above are represented as mean ± SD (n = 5–6). Means with different letters were significantly different. A *p*-value < 0.05 was considered statistically significant.

The protein level of NF-kB in the LF and GFL groups was significantly reduced compared to that in the DMC group. Similar to the result of NF-kB expression, elevated levels of TNF-α and IL-1β were restrained in the GFL and LF groups, respectively, compared to the DMC group. The protein level of IL-6 in the DMC group was higher than that in the NC group, but there was no significant difference among the DM groups (Figure 7).

## 4. Discussion

This study investigated the effects of metabolites derived from *L. fermentum* or from guava leaf fermented by *L. fermentum* on fatty liver in diabetic mice. Major components derived from guava leaf fermented by *L. fermentum* are BCHAs, gallic acid, and glucogallin, according to our previous study [18]. These metabolites are expected to alleviate not only diabetes but also fatty liver through an additive/synergistic effect between them.

The current study found that administration of GFL, as well as LF, decreased FBG and HbA1c levels in diabetic mice. Recent studies showed that *L. fermentum* supplementation alleviated hyperglycemia in diabetic mice [35]. While FBG captures glucose fluctuations over a short period, HbA1c reflects long-term glycemic trends [36]. GFL supplementation led to a greater reduction in HbA1c levels compared to LF supplementation, suggesting that GFL was more effective in glycemic control in diabetic mice. Moreover, GFL alleviated hyperphagia resulting from insulin resistance under hyperglycemic conditions. Similarly, a previous study reported that administration of gallic acid, a phenolic acid present only in GFL, significantly decreased FBG and HbA1c levels in T2DM rats [37]. Additionally, quercetin, a flavonoid present only in GFL, was shown to ameliorate hyperglycemia by enhancing antioxidant enzyme activity in *db/db* mice [38]. These results indicate that *L. fermentum*-derived bacterial metabolites are beneficial for attenuating hyperglycemia in T2DM, but their efficacy can be reinforced and expanded through fermentation with guava leaf.

Ser/Thr phosphorylation of IRS-1 induces insulin resistance by interfering with the tyrosine phosphorylation cascade of insulin signaling [39]. In this study, both LF and GFL supplementation attenuated hepatic insulin resistance demonstrated by reduced p-IRS-1(Ser) to IRS-1 ratio in diabetic mice. Similarly, administration of yogurt containing BCHAs prevented insulin resistance in obese mice [20]. Furthermore, GFL supplementation increased the ratio of p-AKT/AKT in diabetic mice. A prior study indicated that gallic acid attenuated hyperglycemia by improving hepatic carbohydrate metabolism along with lowering hepatic insulin resistance in high-fructose-diet-treated rats [40]. The result demonstrated that enhanced hepatic insulin signaling by GFL supplementation may contribute to lower HbA1c levels in diabetic mice.

In diabetic conditions, hepatic insulin resistance immoderately activates lipogenesis, contributing to fat deposition in the liver [6]. In this study, LF supplementation reduced hepatic TC levels and the percentage of lipid droplets in hepatic tissue. The previous study reported that BCHAs contributed to preventing hepatic steatosis by lowering liver weight and hepatic TG levels in obese mice [20]. On the other hand, the GFL group showed lower hepatic TC and TG levels, as well as the fewest lipid droplets in hepatic tissue among all diabetic groups. In addition, increased liver weight due to a compensatory response to restore damaged hepatic function was reduced only in the GFL group. A previous study demonstrated that gallic acid, contained in GFL, ameliorated hepatic steatosis by normalizing insulin signaling, lipid profile, and liver weight in diabetic rats [41]. Moreover, quercetin, one of the components in GFL, is a well-known hepatoprotective agent in the prevention of MAFLD, acting as an effective free radical scavenger [42]. Considering all these results, it can be inferred that GFL is more effective for mitigating hepatic steatosis than LF. In particular, substances only contained in GFL might further alleviate fatty liver by combining them with the bacterial metabolites derived from *L. fermentum*.

Aberrant hepatic lipid homeostasis characterized by a high influx of lipids and excessive lipogenesis results in hepatic fat deposition and is a hallmark of MAFLD [43]. PPAR-γ regulates the influx of free fatty acids into the liver through fatty acid transporters. Furthermore, it activates SREBP-1c, which activates the expression of genes integral to lipogenesis, including ACC [44,45]. The current study demonstrated that both LF and GFL supplementation suppressed lipogenesis by reducing the expression of key lipogenic transcription factors, PPAR-γ and SREBP-1c, in diabetic mice. In relation to this, *L. fermentum* relieved fatty liver by inhibiting the expression of PPAR-γ, SREBP-1c, CCAAT/enhancer-binding protein-α (C/EBPα) and their downstream targets in obese mice [13]. The results demonstrate that the effect of *L. fermentum* on fatty liver might be partially due to its metabolites.

It is known that abnormal lipid homeostasis is concomitant with diminished energy metabolism in T2DM [10]. Particularly, SIRT1 affects energy homeostasis by regulation of glucose metabolism in hepatic tissue [8]. In contrast, SIRT4 disrupts the interaction between SIRT1 and PPAR-α, negatively regulating mitochondrial function by inhibiting fatty acid oxidation [46]. In the current study, only GFL supplementation significantly improved energy metabolism by activating the SIRT1/PPAR-α/PGC-1α pathway and decreasing the protein level of SIRT4 in diabetic mice. In this line, previous studies have reported that gallic acid attenuated MAFLD by modulating lipogenesis/lipid oxidation and mitochondrial function through the AMPK/ACC/PPAR-α axis [47]. It could be speculated that metabolites found only in GFL may offer valuable assistance in alleviating MAFLD by improving energy metabolism via the SIRT1/PPAR-α/PGC-1α pathway.

Mitochondrial dysfunction and oxidative stress from excess fat deposition trigger a hepatic inflammatory response, contributing to the deterioration of MAFLD [48]. In this study, LF and GFL supplementation decreased NF-kB, a key transcription factor of inflammation, in diabetic mice. This finding indicates that both LF and GFL treatments could prevent liver injury by suppressing NF-kB-mediated inflammation in diabetic mice.

In summary, both GFL and LF supplementation alleviated hyperglycemia and fatty liver in diabetic mice. These effects could be attributed to *L. fermentum*-derived metabolites such as branched-chain amino acids, their catabolites (2-hydroxyisocaproic acid and 2-hydroxyisovaleric acid), and organic acids. Furthermore, enhanced SIRT1-mediated energy metabolism was observed exclusively in the GFL group but not in the LF group. It can be inferred that metabolites only present in GFL might contribute to this difference, although more specific mechanisms of components in GFL need to be investigated [29,42,49,50,51,52,53]. In addition, these findings may stem from potential addictive/synergistic effects among bacterial metabolites and bioactive substances derived from the biotransformation of guava leaf extract (GFL).

## 5. Conclusions

Taken together, the current study showed that GFL has superior efficacy compared to LF in alleviating hyperglycemia and fatty liver by improving hepatic insulin signaling and inhibiting hepatic lipogenesis and inflammation in diabetic mice. Especially, GFL attenuated fatty liver through the improvement of energy metabolism via the SIRT1/PPAR-α/PGC-1α pathway in diabetic mice. In conclusion, GFL containing bioactive substances originating from the co-fermentation process of guava leaf and *L. fermentum* may be utilized as a promising nutraceutical for relieving hyperglycemia induced MAFLD.

## Figures and Tables

**Table 1:** Effects of GFL supplementation on body weight, body composition, food intake, and liver weight in diabetic mice.

Group	NC	DMC	LF	GFL	*p*-Value
Body weight (g)	33.54 ± 3.95	36.11 ± 4.08	34.64 ± 3.23	35.12 ± 3.90	0.71
Body composition (%)					
Fat mass	25.24 ± 3.17	30.96 ± 6.21	26.95 ± 2.96	30.51 ± 4.37	0.11
Lean mass	72.07 ± 3.11	66.65 ± 6.11	70.64 ± 2.95	67.10 ± 4.20	0.12
Food intake (kcal/day)	12.84 ± 0.58 ^a^	14.39 ± 0.65 ^b^	14.77 ± 1.35 ^b^	13.13 ± 0.19 ^a^	0.00
Liver weight (g/g BW)	0.034 ± 0.002 ^a^	0.052 ± 0.008 ^c^	0.045 ± 0.004 ^bc^	0.041 ± 0.007 ^b^	0.00

All values above are represented as mean ± SD (n = 5–6). Means with different letters were significantly different. A *p*-value < 0.05 was considered statistically significant.

## Data Availability

The data presented in this study are available on request from the corresponding author. The data are not publicly available due to privacy. The studies not involving humans.
